# Anti-scattering light focusing by fast wavefront shaping based on multi-pixel encoded digital-micromirror device

**DOI:** 10.1038/s41377-021-00591-w

**Published:** 2021-07-20

**Authors:** Jiamiao Yang, Qiaozhi He, Linxian Liu, Yuan Qu, Rongjun Shao, Bowen Song, Yanyu Zhao

**Affiliations:** 1 Department of Instrument Science and Engineering, School of Electronic Information and Electrical Engineering, Shanghai Jiao Tong University, 200240 Shanghai, China; 2 Shanghai Center for Brain Science and Brain-Inspired Technology, 200031 Shanghai, China; 3 School of Automation and Software Engineering, Shanxi University, 030006 Taiyuan, China; 4 Beijing Advanced Innovation Center for Biomedical Engineering, Key Laboratory for Biomechanics and Mechanobiology of Ministry of Education, School of Engineering Medicine, Beihang University, 100191 Beijing, China

**Keywords:** Imaging and sensing, Biophotonics

## Abstract

Speed and enhancement are the two most important metrics for anti-scattering light focusing by wavefront shaping (WS), which requires a spatial light modulator with a large number of modulation modes and a fast speed of response. Among the commercial modulators, the digital-micromirror device (DMD) is the sole solution providing millions of modulation modes and a pattern rate higher than 20 kHz. Thus, it has the potential to accelerate the process of anti-scattering light focusing with a high enhancement. Nevertheless, modulating light in a binary mode by the DMD restricts both the speed and enhancement seriously. Here, we propose a multi-pixel encoded DMD-based WS method by combining multiple micromirrors into a single modulation unit to overcome the drawbacks of binary modulation. In addition, to efficiently optimize the wavefront, we adopted separable natural evolution strategies (SNES), which could carry out a global search against a noisy environment. Compared with the state-of-the-art DMD-based WS method, the proposed method increased the speed of optimization and enhancement of focus by a factor of 179 and 16, respectively. In our demonstration, we achieved 10 foci with homogeneous brightness at a high speed and formed W- and S-shape patterns against the scattering medium. The experimental results suggest that the proposed method will pave a new avenue for WS in the applications of biomedical imaging, photon therapy, optogenetics, dynamic holographic display, etc.

## Introduction

As photons are propagating through a scattering medium, their trajectories are randomly changed, so they cannot be focused to a micrometer-scale spot, which fundamentally limits the application of optical imaging, photon therapy, and optogenetics^1,2^. By using a spatial light modulator (SLM), wavefront shaping (WS) can manipulate the incident wavefront so that the scattered photons will contribute a constructive interference and form a tight focus against the scattering medium^3,4^. The applications of WS include improving the spatial resolution of biomedical imaging^5,6^, optogenetics^7^, and cataract correction^8^, as well as enlarging the viewing angle in dynamic three-dimensional holography^9^.

Transmission matrix measurement and iterative WS are two methods to implement WS^10^. By measuring the transmission matrix of the scattering medium and solving an inverse problem, the transmission matrix method calculates the optimized incident wavefront to focus the scattered light against the scattering medium^11^. If the optical property of the scattering medium is changed during the measurement, this method will fail and is not useful on highly dynamic scattering media^10,12^. By measuring a feedback signal, the iterative WS method optimizes the incident wavefront step by step and gradually increases the light intensity at the desired focus. The feedback signals can be light intensity^13,14^, fluorescence^5,15,16^, photoacoustic signal^17,18^, etc. This method can adjust the incident wavefront with respect to the current status of the dynamic scattering medium and therefore has the potential to be applied in highly dynamic environments^12,19^.

To achieve high-enhancement light focusing within highly dynamic scattering media, high-speed iterative optimization and a large number of modulation modes are essential for the iterative WS method. On the one hand, a variety of iterative optimization algorithms have been proposed, including the continuous sequential optimization^20,21^, partitioning algorithm^13^, genetic algorithm (GA)^19,22^, particle swarm optimization^23^, Hadamard algorithm^24^, simulated annealing algorithm^25^, etc. Compared with the conventional transmission matrix methods that require an enumerate search, these methods^19–25^ significantly accelerate the process of optimizing the incident wavefront, but the speed of optimization is still not high enough to deal with the high dynamic media. In addition, these methods involve stochastic variables in the optimization and are sensitive to the initial condition and local minima, which constrains the enhancement of the focus. Therefore, it is desirable to have a new optimization method with a high speed and strong capability of global search.

On the other hand, the SLMs must have a high pattern rate and a large number of modulation modes to achieve a high enhancement of the focus against highly dynamic perturbations in the scattering medium. The SLMs can be categorized into three groups: continuous phase modulation^26–31^, binary amplitude modulation^32–34^, and polarization modulation^35^. Liquid crystal SLM (LC-SLM) is the most common choice for continuous phase modulation^29–31^. They generally have millions of pixels modulating the phase of the wavefront quasi-continuously. However, the typical pattern rate of LC-SLM is ~30 Hz, which is too slow to accelerate the iterative optimization. Within the group for binary amplitude modulation, microelectromechanical grating light valves have a high pattern rate (350 kHz) and can focus the light in milliseconds^32^; but, they have only ~1 k pixels, which compromises the enhancement of the focus. In contrast, digital-micromirror devices (DMD) take advantage of a high pattern rate and a large number of pixels^33,34^. For example, the DMD made by Texas Instruments (DLP9500) has two million pixels and a 23 kHz pattern rate, but the DMD modulates the light amplitude in a coarsely discretized binary manner, which slows down the speed of searching the optimized incident wavefront and degrades the enhancement of the focus. Therefore, methods that can circumvent these problems caused by the inflexible light modulation will convert DMD into an ideal device for anti-scattering light focusing.

Here, we propose a multi-pixel encoded DMD (mpDMD)-based WS method that combines multiple pixels in the DMD into a single modulation mode and encodes the multiple pixels together to solve the problems caused by the coarsely discretized modulation. The binary amplitude modulation in which the amplitude difference between two neighboring code values could vary dramatically degrades the modulation accuracy. Conversely, with a smooth change between consecutive code values, the proposed encoding strategy can guarantee a better accuracy. In addition to the new DMD encoding method, we adopted separable natural evolution strategies (SNES) to considerably accelerate the global search for iteratively optimizing the wavefront. We studied the optimized number of pixels for encoding and established a double-pixel encoding strategy to achieve the highest enhancement of the foci. We demonstrated that, compared with the conventional method modulating light with independent micromirrors, the proposed method increased the speed of optimization and the enhancement of foci by a factor of 179 and 16, respectively. Finally, we illustrated the potential of this method by optimizing the brightness of 10 foci simultaneously and forming W- and S-shape patterns in the noisy background.

## Results

### Principle of the mpDMD-SNES WS

**Fig. 1:**
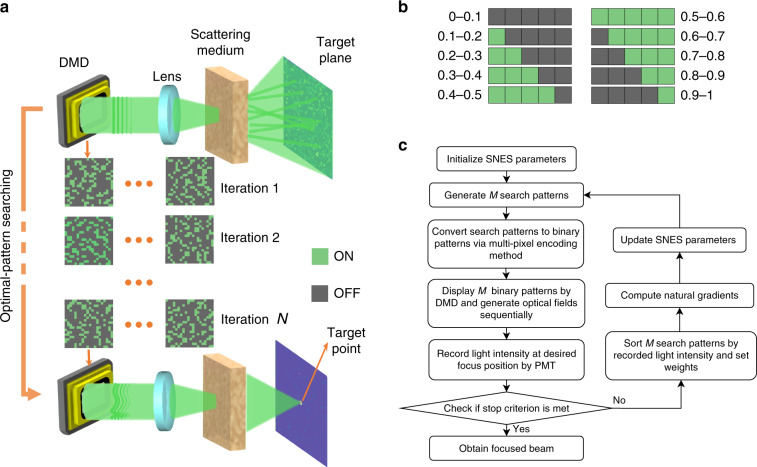
Principle and schematic of mpDMD-SNES WS method. **a** Schematic of the iterative WS method; **b** Code of the multi-pixel DMD encoding strategy, where the number of encoded pixels is 5; **c** Diagram of SNES.

As shown in Fig. 1, the proposed mpDMD-SNES WS method used the light intensity at the target point as feedback, treated multiple micromirrors as a single modulation mode to modulate the amplitude profile of incident light, and applied SNES to adjust the displayed pattern in the DMD to optimize the incident wavefront. As a result, the light transmitted through the scattering medium was gradually focused onto the target point with a high enhancement over a short optimization period.

Since each micromirror in the DMD modulates light independently, we set the Hamming distance between the neighboring code values to 1 in the multi-pixel encoding process to mimic a continuous modulation on the light amplitude. As shown in Fig. 1b, a single modulation mode with *n*_bin_ micromirrors encoded a real number *x* between 0 and 1. The state of the *i*th (1 ≤ *i* ≤ *n*_bin_) micromirror could be calculated by Eq. (1):

1 $$g_i\left( x \right) = \left\{ {\begin{array}{*{20}{c}} {1,} & {x \in \left[ {c_i - l,\;c_i + l} \right]} \\ {0,} & {{\mathrm{else}}} \end{array}} \right.$$

where 1 is the ON state and 0 is the OFF state; $c_i = \frac{{n_{{\mathrm{bin}}} + 2i}}{{4n_{{\mathrm{bin}}}}};\;l = 1/4$.

Figure 1c shows how the SNES optimizes the incident wavefront. The SNES iteratively searches and updates the Gaussian parameters along the direction of the natural gradient. The Gaussian’s parameters allow the SNES to adaptively capture the structure of the feedback function. As a result, these parameters can be adjusted with respect to the amplitude of the feedback, and the incident wavefront is optimized. The natural gradient provides a direction along which the feedback amplitude increases as well, and it can prevent the oscillation in the process of convergence and escape the traps of local minima.

Given that the number of pixels in the DMD is *N*, the number of modulation modes is *N*_s_ = *N*/*n*_bin_. During optimization, the modulation amplitude of each mode is parameterized by the Gaussian parameters *µ* and *σ*. The *M* initial states for each mode are set according to the Gaussian function with *µ* *+* *σs*_*m*_. Here, *s*_*m*_ (*m* = 1, 2, …, *M*) is a search point with a dimension of *N*_s_, and its entries follow the standard normal distribution. Each search point corresponds to a particular binary pattern in the DMD. A photomultiplier tube (PMT) at the target point records the light intensity (*I*_1_, *I*_2_…*I*_M_) with respect to each DMD pattern as the feedback for the SNES. We can sort the search points according to their feedback amplitudes in increasing order and multiply with weights *u*_*m*_. The weights for the first *M*/2 search points are set to 0. For the rest of the weights as an arithmetic sequence, we can set their sum to 1. The natural gradients $\nabla _\mu J$ and $\nabla _\sigma J$ for the mean *µ* and standard deviation *σ* respectively can be calculated by Eq. (2).

2 $$\left\{ {\begin{array}{{ll}} {\nabla _\mu J = \mathop {\sum}\nolimits_{{\mathrm{m = 1}}}^M {u_m \cdot s_m} } \\ {\nabla _\sigma J = \mathop {\sum}\nolimits_{{\mathrm{m = 1}}}^M {u_m \cdot \left( {s_m^2{\mathrm{ - 1}}} \right)} } \end{array}} \right.$$

The optimization program updates *µ* and *σ* using Eq. (3).

3 $$\left\{ {\begin{array}{*{20}{c}} {\mu _{{\mathrm{i}} + {\mathrm{1}}} = \mu _i + {\it{\upeta }}_\mu \sigma _i \cdot \nabla _\mu J} \\ {\sigma _{{\mathrm{i + 1}}} = \sigma _i{\mathrm{exp}}\left( {\frac{{\eta _\sigma }}{2}\nabla _\sigma {\mathrm{J}}} \right)} \end{array}} \right.$$

Here, *η*_*μ*_ and *η*_*σ*_ are the learning rates for *µ* and *σ*, respectively. In each iteration, the program generates *M* new DMD patterns to efficiently search the best modulation focusing the light on the target point. The SNES needs only the Gaussian parameters rather than an entire memory space that saves the state of each mode for each search point. Therefore, the SNES is faster due to the less memory cost than the conventional methods.

### Performance verification by numerical simulation

To verify the performance of the mpDMD-SNES-based WS, we first carried out a numerical simulation. The number of pixels was *P* for the camera and *N* for the DMD. The target point was the center of the camera. The transmission matrix from the DMD in the initial state through the scattering medium to the camera was *T*. We assumed that the scattering medium was totally random so that each entry *t*_*pn*_ in *T* followed an independent Gaussian distribution^36^. Then, we obtained the light intensity at the pixel *p* of the camera^20^

4 $$I_p = \left|\mathop {\sum}\nolimits_{n = 1}^N {t_{{pn}}A_n}\right| ^2$$

where *A*_*n*_ was the amplitude of the pixel *n* of DMD and could be 0 or 1. As DMD modulated the amplitude only, we neglected all phase information.

**Fig. 2:**
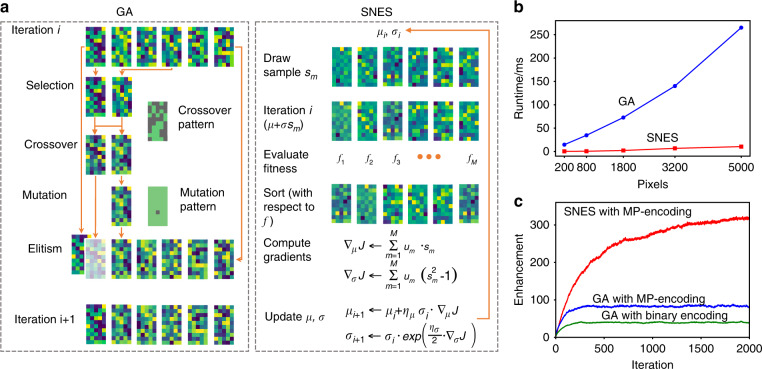
Comparison of GA and SNES by numerical simulation. **a** Diagrams of GA (left) and SNES (right). **b** Computation times of the two algorithms. **c** Enhancement of the light intensity at the target point. MP, multi-pixel.

Figure 2a shows the diagrams of SNES and GA to implement the WS. In the simulation, we set *n*_bin_ = 5 and *M* = 30. As the feedback optimizing the light intensity at the target point, the enhancement was defined as the mean ratio of the intensity of the focus to the intensity of the scattered light before optimization. The parameters of the SNES and GA are detailed in Methods section and both had optimized setting obtained based on literature^37^. We also analyzed the computation times of SNES and GA with respect to *N*_s_ after an average over 10,000 iterations (Fig. 2b). Both algorithms were run on a GPU. When the number of modulation modes in the DMD increased from 200 to 5,000, the computation times of SNES and GA increased from 0.38 ms and 14.58 ms to 10.42 ms and 265.10 ms, respectively. Comparing with the GA, the SNES reduced the computation time by a factor of 25. Finally, we studied the enhancement as a function of iterations in various architectures, including the GA with binary encoding, GA with multi-pixel encoding, and SNES with multi-pixel encoding. The corresponding enhancements were 43, 89, and 322, respectively. The enhancement was increased by a factor of 7.5 after the architecture was transformed from the GA with binary encoding to the SNES with multi-pixel encoding.

### Experimental set-up and characterization

**Fig. 3:**
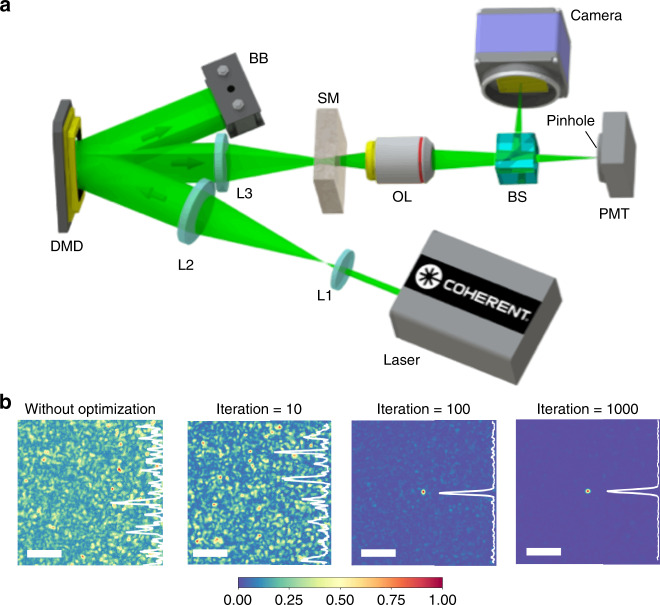
Demonstration for the DMD-SNES-based WS. **a** Setup of the experiment. L, lens; DMD, digital-micromirror device; BB, beam blocker; SM, scattering medium; OL, objective lens; BS, beam splitter. **b** Normalized intensity distribution of the images captured by the camera before the optimization, and after 10, 100, and 1000 iterations (from left to right). The white curve on the right-hand side of each image shows a normalized light intensity distribution along the central vertical line. Scale bar, 500 µm.

Our WS system based on multi-pixel DMD encoding is shown in Fig. 3a. A collimated light beam from a 532 nm laser went through a beam expander (combined with lenses L1 and L2) and was reflected by a DMD. The iterative SNES method generated DMD patterns to modulate the amplitude profile of the light. The modulated light beam was focused by a lens L3, passed through a ground glass diffuser, and was projected by an objective lens. A beam splitter was in the front of the objective lens to divide the scattered beam into two. One beam was pointed towards a PMT behind a pinhole with a diameter of 50 µm at the target point for feedback; the other was pointed towards a camera conjugate to the pinhole, monitoring the image of the focus to calculate the enhancement. During the initialization, we captured 10 images by randomly manipulating the DMD, to find out the initial intensity of the focus, by averaging the values detected by the PMT over the ten images, which mitigated the stochastic effect of scattered light on the value of the enhancement.

Figure 3b illustrates the process of anti-scattering light focusing when *n*_bin_ = 2, by showing the images of the focus before the optimization, and after 10, 100, and 1,000 iterations. The images’ corresponding enhancements were 1, 6.29, 20.64, and 70.89, respectively. Without any optimization, the focus at the center was overwhelmed in the background. As the number of iterations increased, the focus gradually got stronger. After 1000 iterations, the randomly scattered spots almost disappeared in the image.

**Fig. 4:**
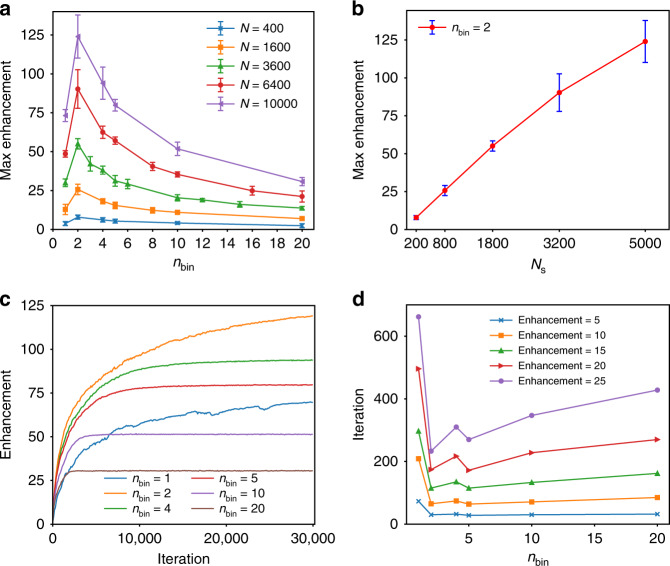
Optimization curves in terms of *n*_bin_ and *N*_s_. **a** Relations between the maximum of enhancements and *n*_bin_, if the number of DMD pixels *N* are 400, 1600, 3600, 6400, and 10,000, respectively. *n*_bin_ = 1 represents the binary encoding. **b** Maximum of enhancements as a function of the number of modulation modes when *n*_bin_ = 2. Standard deviation over 10 data sets was indicated by the error bars. **c** Optimization curves with respect to various *n*_bin_ when *N* = 10,000. **d** Relations between the number of iterations to obtain a certain enhancement and *n*_bin_. We chose the enhancements equal to 5, 10, 15, 20, and 25 for demonstration. *N* = 10,000.

We also tested the effect of *n*_bin_ and *N*_s_ on the intensity of the focus, as shown in Fig. 4. All the data were averaged by 10 repetitions to reduce the randomness. The results first revealed that the maximum enhancement did not increase monotonously with *n*_bin_ when the number of pixels *N* is fixed (Fig. 4a). While *n*_bin_ (>2) was increasing, the maximum enhancement was decreasing, because the larger *n*_bin_ had a trade-off with the *N*_s_ in the DMD for a higher encoding accuracy. Figure 4b illustrates the relation between the maximum of enhancements and *N*_s_ when *n*_bin_ = 2. The more modulation modes the DMD had, the larger the maximum was. We also found that the intensity of the focus increased at the fastest speed when *n*_bin_ = 2, from the optimization curves (Fig. 4c, d). Therefore, in the following experiments, we set *n*_bin_ = 2 and *N*_s_ = 5000.

### Comparison between mpDMD-SNES and state-of-the-art WS method

**Fig. 5:**
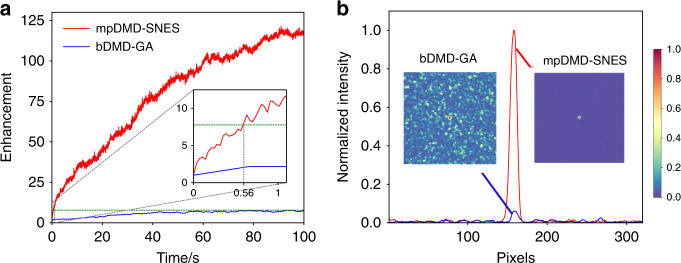
Experimental comparison between mpDMD-SNES and bDMD-GA WS methods. **a** Optimization curves of mpDMD-SNES and bDMD-GA WS methods. The inset zooms in the interval between 0 and 1 s. **b** Light intensity along the central horizontal line in the images of the focus, optimized by mpDMD-SNES (red curve and right image) and bDMD-GA (blue curve and left image) WS methods, respectively. Scale bar, 500 µm.

We further compare our mpDMD-SNES WS method with the state-of-the-art, i.e., the binary-encoding DMD based, GA optimized (bDMD-GA) WS method, in terms of the enhancement and optimization speed (Fig. 5). The mpDMD-SNES WS method in the first 100 seconds kept improving the enhancement to 124, which was increased by a factor of 16 in comparison with the result of the bDMD-GA WS method (Fig. 5a). Setting the number of search points *M* = 30, one iteration of the mpDMD-SNES WS method took 35 ms, in contrast to 670 ms using the bDMD-GA WS method. The green dash line in Fig. 5a represents an enhancement of 7.76. The mpDMD-SNES WS method obtained this value after 0.56 s, faster by a factor of 179 than the bDMD-GA WS method spending 100 s. We also compared the images of the focus (Fig. 5b), optimized by two methods after 100 s. We observed a focus almost overwhelmed in the background by bDMD-GA WS method. Conversely, the focus by the mpDMD-SNES WS method was very bright and obvious.

**Fig. 6:**
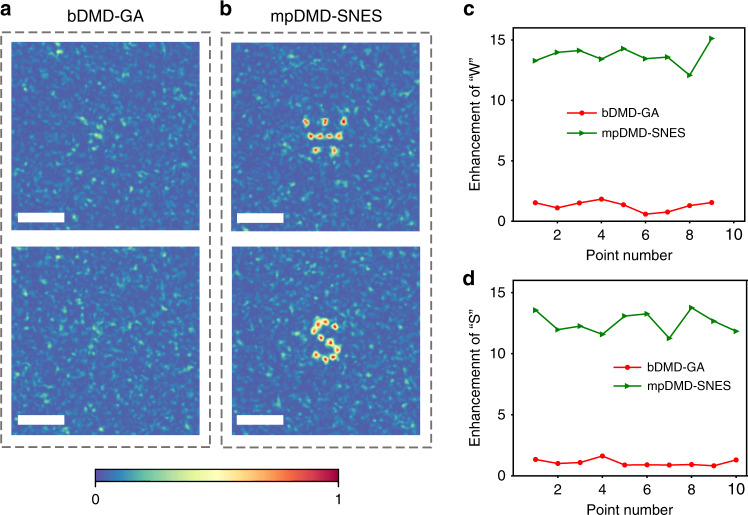
Multi-focus control experiments. **a**, **b** Images of the foci optimized by the bDMD-GA (**a**) and the mpDMD-SNES WS methods (**b**). Scale bar, 500 µm. **c**, **d** Enhancement for each focus in the W- (**c**) and S-shape (**d**) patterns.

In the end, we achieved and demonstrated multi-focus control with homogeneous brightness and formed W- and S-shape patterns against the scattering medium. The images of the foci after 3,000 iterations are shown in Fig. 6a for the bDMD-GA and Fig. 6b for the mpDMD-SNES WS methods. In addition, we compared the normalized intensity of each focus in the W- (Fig. 6c) and S-shape (Fig. 6d) patterns between the two methods. On one hand, the enhancement of the obscure foci optimized by the bDMD-GA WS method was quite low. On the other hand, the mpDMD-SNES WS method could project clear W- and S-shape patterns with homogeneous brightness and provide a way to homogeneously focus the scattered light onto multiple target points. The deviation of focal enhancements in the W- and S-shape patterns are 0.79 and 0.83, respectively, about 6% with respect to the mean enhancement in each pattern.

## Discussion

We have proposed mpDMD-SNES WS that is an iterative method and encodes multiple pixels of DMD to focus light against scattering media over a short optimization period. Encoding multiple neighboring pixels in the DMD enables a quasi-continuous light amplitude modulation, circumventing the obstacles created by the conventional binary amplitude modulation, which unambiguously improves the competitiveness of DMD for WS. Moreover, SNES as a global search algorithm has a faster speed to figure out the optimal DMD modulation than the other conventional methods like the “half-blind” GA method^19–25^. In our experiments, we compared the mpDMD-SNES WS method with the state-of-the-art bDMD-GA method. The mpDMD-SNES method could improve the enhancement of the focus and the speed of optimization by a factor of 16 and 179, respectively. This method furthermore achieved homogeneously focusing 10 spots against the scattering medium over a short optimization period and forming W- and S-shape patterns standing out from the noisy background.

The mpDMD-SNES WS method still has the space to be improved. The current limit on the optimization speed originated from the communication among devices and the computing power of the hardware. The light intensity recorded by the PMT as the feedback was digitized and transferred to the computer. After one iteration of optimization, the computer sent a new pattern to the DMD. This multi-step communication arrangement limited the speed of the optimization process, and consequently one iteration took 35 ms. If the feedback detector is directly connected to a SLM, with the help of a field programmable gate array (FPGA) running the optimization program, the runtime of one iteration could approach 30 µs, achieving the speed limit ultimately set by the maximum pattern rate of DMD. In other words, the process of optimization could be accelerated by three orders of magnitude.

We note that the mpDMD-SNES WS method is compatible with fluorescent markers^38^, photoacoustic feedback^39^, and a variety of other WS guide stars^18,40,41^. Their integration perhaps will surmount the difficulties caused by the perturbation of physiological motions in tissues and extend their capability to high-enhancement light focusing in deep tissues. In addition, mpDMD-SNES without guide stars still can be useful for many applications, such as holographic display^15^, in which manipulating light behind a scattering medium could help achieve an extremely large viewing angle and image size at the same time. Our work puts a new perspective on light focusing through or within dynamic scattering media and promises various applications, including optogenetics, photon therapy, and dynamic holographic display, etc.

## Materials and methods

### Details of the numerical simulation

The numerical simulation was conducted on a GPU (GeForce GTX 1660 Ti, NVIDIA). The SNES had parameters of *η*_*μ*_ = 1 and *η*_*σ*_ = 0.039, while the GA had crossover rate of 85%, mutation rate of 10% and elite rate of 20%, which were determined from literature for optimal performance^37^. The population size for SNES and GA are both 30. The simulations were repeated 10 times to reduce randomness.

### Experiment system

The system diagram is shown in Fig. 3a. The illumination source was a continuous-wave 532 nm laser (Verdi V5, Coherent, Inc.), producing collimated light beam for illumination. The amplitude modulator is a digital-micromirror device (DMD) with pixel size of 1024 × 768 (Texas Instruments, Inc.). A ground glass diffuser (DG10-120, Thorlabs, Inc.) was used as scattering medium, followed by an objective lens (LA1765-A, Thorlabs, Inc.). A beam splitter (BS004, Thorlabs, Inc.) was placed after the scattering medium to divide the scattered beam and relay onto the PMT (H10721-20, Hamamatsu) and the camera (PCO.edge 5.5, PCO, Corp.), respectively. The camera was located at the conjugate position of the PMT to monitor the image of the generated focus. A number of 10 patterns was generated and displayed on the DMD with corresponding images and PMT values collected to evaluate the initial focus intensity.

## Data Availability

The relevant data supporting the findings of this study are available within the paper and from the corresponding author upon reasonable request.

## Code availability

The relevant scripts supporting this study are available from the corresponding author upon reasonable request.
